# Extracts of Poplar Buds (*Populus balsamifera* L., *Populus nigra* L.) and Lithuanian Propolis: Comparison of Their Composition and Biological Activities

**DOI:** 10.3390/plants10050828

**Published:** 2021-04-21

**Authors:** Monika Stanciauskaite, Mindaugas Marksa, Mindaugas Liaudanskas, Liudas Ivanauskas, Marija Ivaskiene, Kristina Ramanauskiene

**Affiliations:** 1 Department of Clinical Pharmacy, Faculty of Pharmacy, Lithuanian University of Health Sciences, Sukileliai Avenue 13, LT-50161 Kaunas, Lithuania; Kristina.ramanauskiene@lsmuni.lt; 2 Department Analytical & Toxicological Chemistry, Faculty of Pharmacy, Lithuanian University of Health Sciences, Sukileliai Avenue 13, LT-50161 Kaunas, Lithuania; mindaugas.marksa@lsmu.lt (M.M.); liudas.ivanauskas@lsmuni.lt (L.I.); 3 Department of Pharmacognosy, Faculty of Pharmacy, Lithuanian University of Health Sciences, Sukileliai Avenue 13, LT-50161 Kaunas, Lithuania; mindaugas.liaudanskas@lsmuni.lt; 4 Dr. L. Kriauceliunas Small Animal Clinic, Veterinary Academy, Lithuanian University of Health Sciences, Tilzes str. 18, LT-47181 Kaunas, Lithuania; marija.ivaskiene@lsmuni.lt

**Keywords:** balsam poplar, black poplar, propolis, phenolic compounds, flavonoids, antioxidant activity, antimicrobial activity

## Abstract

Balsam poplar and black poplar (*Populus balsamifera* L. and *Populus nigra* L.) buds that grow in Lithuania are the primary source of propolis, therefore it is proper to evaluate and compare the composition of these raw plant materials and propolis quantitatively and qualitatively. Propolis and balsamic poplar bud extract are dominated by *p*-coumaric acid and black poplar-caffeic acid. Antioxidant activity was evaluated by DPPH (2,2-diphenyl-1-picrylhydrazyl), ABTS (2,2-azino-bis(3-ethylbenzothiazoline-6-sulfonic acid), FRAP (ferric-reducing antioxidant power) and CUPRAC (cupric reducing antioxidant capacity) methods and all extracts showed antioxidant activity, and obtained results correlated with the obtained amounts of phenolic compounds and flavonoids in the extracts. Studies of antimicrobial activity have shown that all extracts have a growth inhibitory effect against *Staphylococcus aureus* and *Candida albicans*, but the extract of balsam poplar buds showed the most significant effect of such kind. Considering the results of the research, it can be stated that balsam poplar buds cultured in Lithuania are the primary raw material of propolis, which is rich in phenolic compounds with antioxidant properties and is a promising raw material for pharmaceutical purposes.

## 1. Introduction

Propolis is a natural substance, widely discussed in scientific literature. Propolis is also a dark resin substance, which has many different compounds: 25–30% of wax, 50–65% of resin, 10% of essential oils and 5% of other compounds [1], and the biologically active agents that are most abundant in it [2] are flavonoids and phenolic acids. The chemical composition of propolis is closely connected with and dependent on raw plant material, which is collected by bees in different regions of the world, therefore its chemical composition may differ [1,3]. In temperate climate zones, the main source of wax for bees is poplar buds. In Europe, Northern America and Australia, poplar buds are the main raw material used by bees to collect resin [4]. Scientific research was carried out, and it was noticed that the main components in poplar buds were phenolic acids and flavonoids [5]. Though it is known that poplar buds are the main source of propolis, this raw plant material has been investigated more closely only recently, therefore the scientific literature on it is scarce. There are some comparative data in the scientific literature about propolis and black poplar buds’ chemical composition [6].

De Marco et al. compared biologically active components of poplar buds and Italian propolis and evaluated the total quantity of flavonoids, such as chrizine, galangin and pinocembrin, and they separately evaluated the quantity of caffeic acid phenethyl ester (CAPE) [6]. These compounds are responsible for the antioxidant activity of the raw material. On the basis of scientific research, the scientists claim that poplar buds can be a very good substitute for propolis [7]. 

Since ancient times, propolis has been widely used in folk medicine as raw material for maintaining strong immunity, as it has anti-inflammatory [8], antioxidant [9] and antimicrobial activities [10]. In the long run, scientists started investigating the biological properties of propolis in more detail and noticed that propolis can also have antitumor, anticancer, neuroprotective and other activities [11,12,13]. Nowadays, propolis is widely used in biopharmacy as a supplement or as a cosmetic component for its antioxidant, anti-inflammatory and antimicrobial properties [12,14,15]. While in search of scientifically based usage of propolis, scientists are increasingly examining factors (climatic conditions, plants in the places of collection) which have influence on the chemical composition and biological activity of propolis [16]. 

Since ancient times, poplar bud decoctions and extracts were used for wound healing, alleviation of dermatitis symptoms, treatment of rheumatism and infections of the upper respiratory tract [17]. Black and balsam poplars (*Populus balsamifera* L. and *Populus nigra* L.) [18] are most widely used for curative purposes, but in comparison with propolis, there are few scientific studies on the biological activity of this raw material [12,18]. In vitro comparative studies of antimicrobial activity of different poplar species are presently underway, because the activity of propolis depends on its chemical composition and is different in different countries [5]. Turkish scientists have determined that propolis from poplar buds and poplar buds themselves inhibit clinically significant microorganisms, among them yeast, but they have no impact on Gram-negative bacteria [16,19]. For this stage of the study, *S. aureus* and *C. albicans* were selected. *C. albicans* and *S. aureus* are among the major pathogens in the human body that cause adverse inflammatory reactions [20]. *C. albicans* mainly develops in the mouth and throat area, causing candidiasis, and yeast can affect the cornea of the eye [21]. *S. aureus* is one of the pathogens most commonly causing infectious diseases of various skin and soft tissues, especially when obstacles on the skin or mucosa have been damaged [20]. 

Polish scientists described and evaluated the composition of propolis and black poplar extracts, where the chemical composition of propolis was reflected in the analysis of *P. nigra* composition. The composition of propolis samples is similar to that of the mixture of poplar bud (flavonoids) substances [22]. So far, no analysis has been done on the chemical composition of Lithuanian poplar buds and their biological activity. It is necessary to compare the chemical composition and biological activity of Lithuanian propolis and poplar buds. There are balsam and black poplar trees in Lithuania, slightly less of the latter. During this research, buds of both balsam and black poplar trees and propolis were collected in Lithuania in 2020 and investigated. The results showed that separation of active compounds from the raw material depended on the conditions of extraction and the chosen solvent [23,24]. One of the most effective modern methods of extraction is the separation of the active compounds from the raw material using ultrasound [25]. The most often used solvent in the production of propolis extracts is 70% ethanol, because it is very effective in separation of active compounds from the raw material [26]. Phenolic compounds often dissolve easier in organic solvents than in water [27]. Therefore, with the aim to investigate and compare the quality of the poplar bud extracts with the propolis extracts, we chose 70% ethanol for their production. The aim of this research is to compare qualitative and quantitative composition of active compounds in the produced poplar and propolis extracts, and to determine their antioxidant and antimicrobial activity in vitro. 

## 2. Results

### 2.1. Quality Results

Extract N1 and extract N2 were produced under the same conditions as extract N3. The quality results of the prepared extracts are presented in Table 1.

The results of the study presented in Table 1 show that the total amount of phenolic compounds in the extracts is higher compared to the total amount of flavonoids. The data presented show that the total content of phenolic compounds in the extracts was highest in the balsam poplar buds extract (N1) compared to the extracts N2 and N3. There was a statistically significant difference between the determined amounts of phenolic compounds in the tested extracts (*p* < 0.05). Total flavonoids were also found to be highest in extract N1. The lowest content of flavonoids was determined in extract N3, and the lowest amount of total phenolic compounds was detected in extract N2. The total amount of flavonoids in the extracts was statistically significantly different (*p* < 0.05).

### 2.2. Distribution of Active Compounds

**Figure 1:**
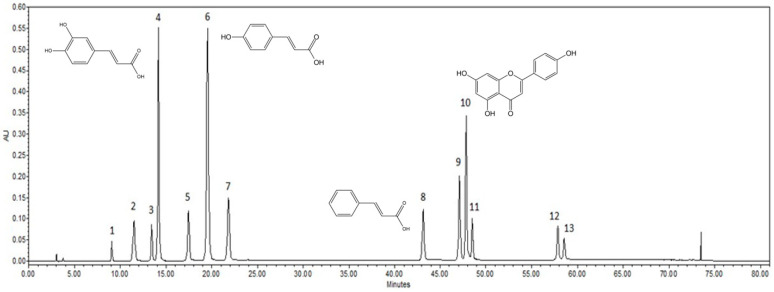
HPLC chromatogram of standards of compounds identified in the extracts: 1. salicin, 2. chlorogenic acid, 3. vanillic acid, 4. caffeic acid, 5. vanillin, 6. *p*-coumaric acid, 7. ferulic acid, 8. cinnamic acid, 9. pinobanksin, 10. apigenin, 11. kaempferol, 12. pinocembrin, 13. galangin.

The chemical composition of the prepared poplar and propolis extracts was evaluated by high-performance liquid chromatography (HPLC), identifying the amounts of phenolic acids, flavonoids, vanillin and salicin. Pure ethanol extracts were used for HPLC analysis without further processing. A typical chromatogram of active compounds is provided in Figure 1.

The research results showed (Table 2) that *p*-coumaric, caffeic and ferulic acids, vanillic acid, pinobanksin, apigenin and galangin were present in analyzed extracts. *P*-coumaric acid was dominant in balsam poplar bud and propolis extracts. The largest amount of caffeic acid was found in black poplar bud extract. Chlorogenic acid was found in poplar bud extracts but it was not found in propolis. The amounts of vanillic and ferulic acids found in propolis were larger than in poplar bud extracts. An exceptionally large amount of cinnamic acid was found in balsam poplar extract. The differences between the quantities of phenolic acids in analyzed extracts were statistically significant (*p* < 0.05). The results demonstrated that galangin was the dominant flavonoid in balsam poplar extract. Comparatively small amounts of galangin were also found in other extracts. The largest amount of pinocembrin was found in the extract of black poplar. The data demonstrated that pinocembrin was not detected in propolis extract. A significant variation of flavonoid quantity was determined in the analyzed extract samples. No vanillin was found in balsam poplar bud extract samples. In black poplar bud extract, the amount of vanillin was insignificant. Meanwhile, in propolis extract, vanillin was determined as one of the dominant active compounds. Salicin was identified in both poplar extracts that were analyzed. The differences in salicin amounts in both extracts were statistically significant (*p* < 0.05). Table 2 demonstrate that a larger amount of salicin was determined in black poplar bud extract compared to balsamic poplar bud extract, while salicin was not detected in the propolis extract.

**Figure 2:**
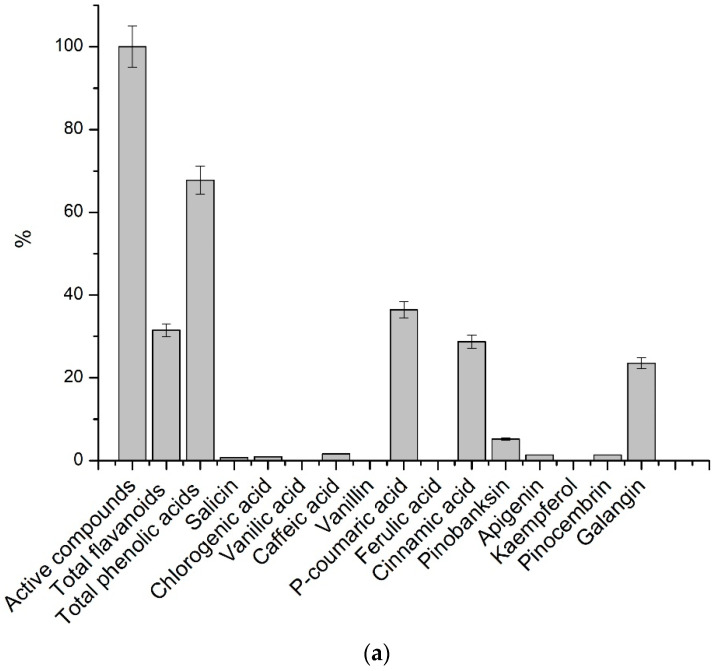

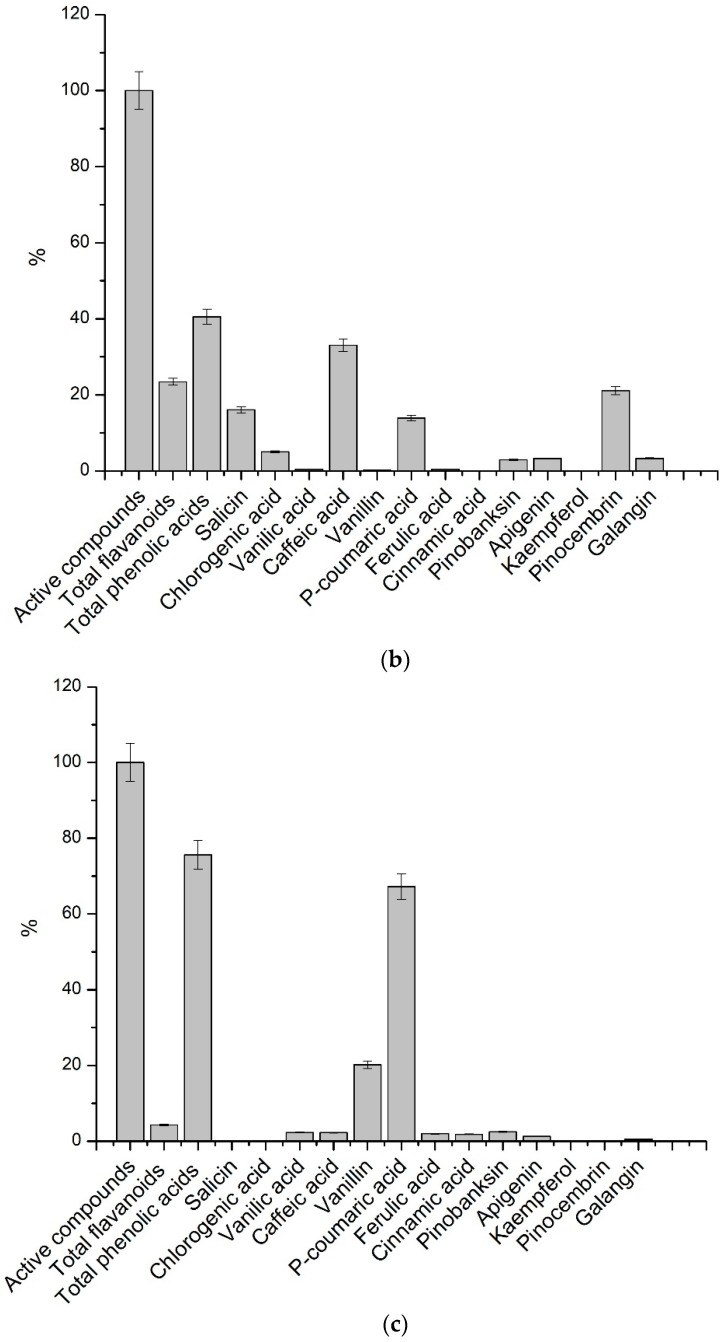
Percentage of active compounds for the identification of test samples out of the total active compounds. (**a**) N1—balsam poplar buds extract, (**b**) N2—black poplar buds extract, (**c**) N3—propolis extract.

The content of detected flavonoids in the analyzed extracts was lower compared to the content of phenolic compounds. Comparison of relative quantities of active compounds in different analyzed extracts demonstrated significant variations of their quantitative composition (Figure 2). In propolis (N3) and balsam poplar bud extract, *p*-coumaric acid accounted for the largest fraction, around 67.1% ± 3.08% and 36.4% ± 1.82% of active compounds. Cinnamic acid was one of the predominant acids in the balsam poplar bud extract, accounting for about 28.7% ± 1.14% of the total active compounds. Caffeic acid accounted for the largest proportion of total active compounds, about 33.07% in black poplar extract. The main flavonoids in the analyzed extracts were galangin and pinocembrin. The highest content of galangin was found in the extract of balsam poplar buds N1, which accounted for about 23.5% ± 1.12% of the total amount of active compounds. In black poplar bud extract N2, pinocembrin comprises approximately 21.13% ± 0.86% of the total amount of active compounds. A larger amount of vanillin was found in propolis extract, but in black poplar bud extract, only traces of vanillin were noticed. The amount of salicin in extract N2 comprises around 16.1% ± 0.65% of the total amount of active compounds. The results of our study show that the chemical composition of the extracts depends on the chosen poplar species. The comparative analysis of poplar bud extracts with propolis extract shows that relative analysis of active compounds is an important indicator in the evaluation of the botanical origin of propolis. 

### 2.3. Antioxidant Activity

**Figure 3:**
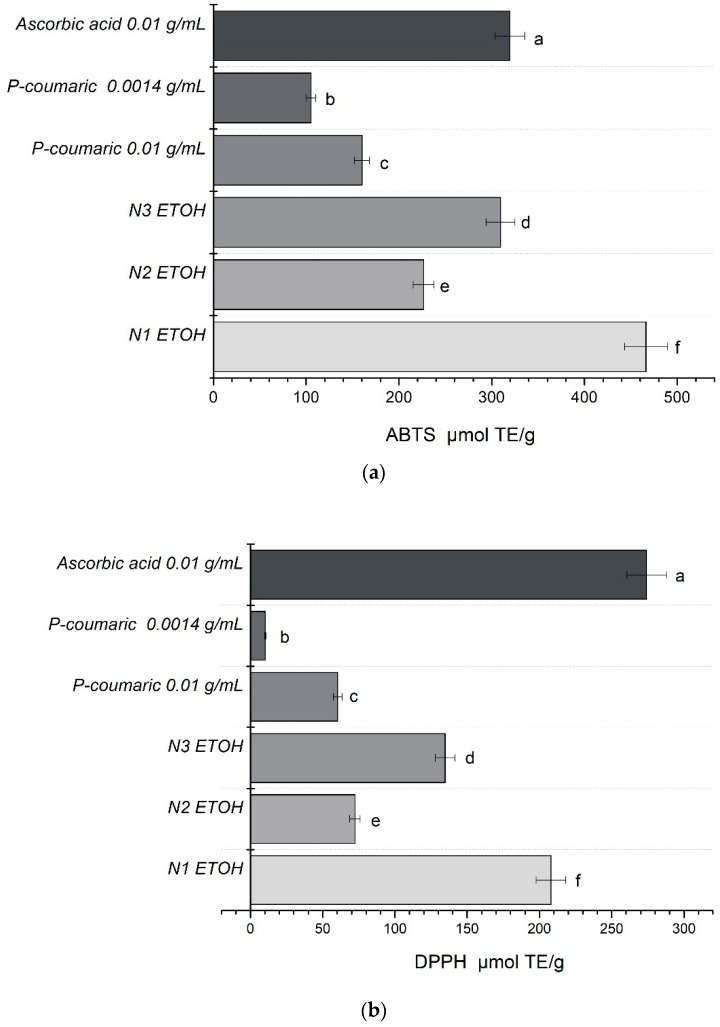

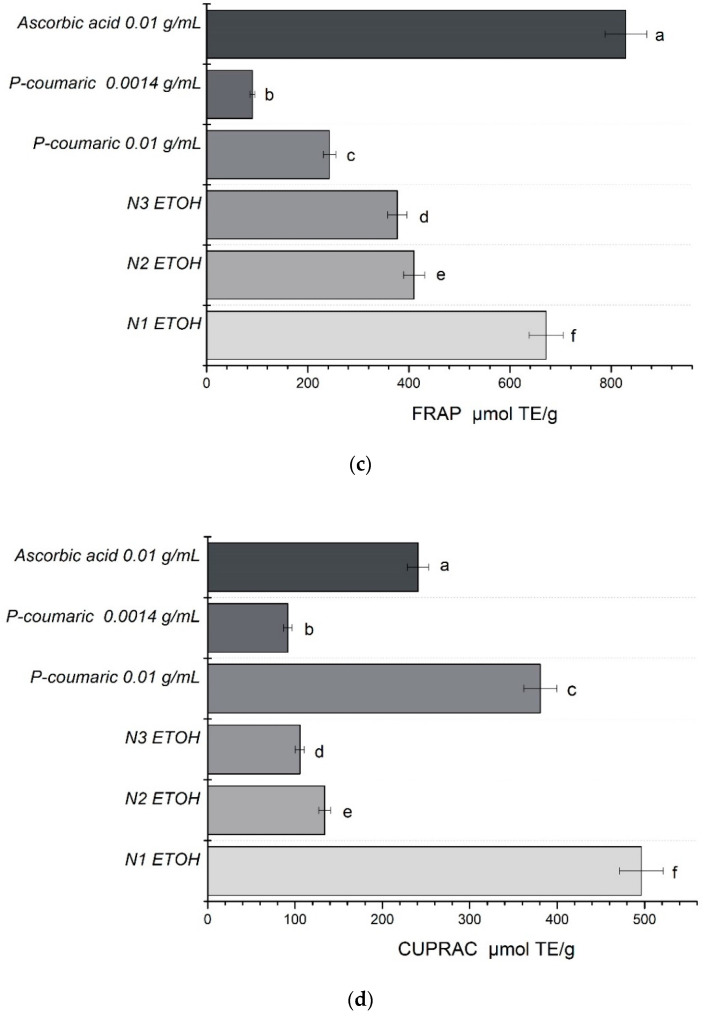
The antioxidant activity of samples N1 (*P. balsamifera* buds extract), N2 (*P. nigra* buds extract) and N3 (Propolis extract) expressed as µmol TE/g. (**a**) ABTS, (**b**) DPPH, (**c**) FRAP, (**d**) CUPRAC. Different letters mean significant difference at *p* < 0.05.

The antioxidant activity of the extracts prepared in the experimental study was analyzed using ABTS, DPPH, FRAP and CUPRAC methods. The research results are presented in Figure 3.

The data presented in Figure 3 show that all extracts have antioxidant activity. *P*-coumaric acid solution had weaker antioxidant activity in comparison with other analyzed extracts, except when the CUPRAC method was applied. When DPPH and FRAP methods were applied, the extracts showed statistically significantly weaker antioxidant activity in comparison with the ascorbic acid solution (*p* < 0.05). When ABTS and DPPH methods were applied, extract N2 had the weakest antioxidant activity. When the antioxidant activity of different poplar bud extracts was compared with the propolis extract, it was noticed that extract N1 had the strongest antioxidant activity. 

A statistically significant difference was determined between the antioxidant activity study results of the analyzed extracts (*p* < 0.05) when FRAP and CUPRAC methods were applied. In the study results, we noticed that extract N1 showed the best antioxidant activity results in comparison with extracts N2 and N3. The analysis of the study results showed that extract N1 had significantly stronger activity (*p* < 0.05) than the antioxidant activity of extracts N2 and N3. 

**Figure 4:**
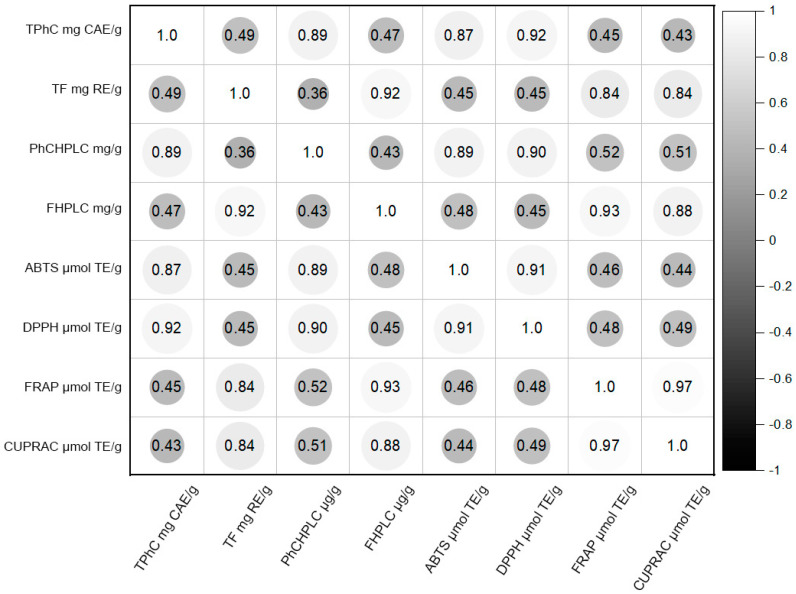
Correlation graph of total phenolic compounds, flavonoid content and antioxidant activity results (TPhC—total phenolic compounds mg CAE/g, TF—total flavonoids mg RE/g, PhHPLC—total phenolic compounds by HPLC mg/g, FHPLC—total flavonoids by HPLC mg/g).

The results of the total phenolic compounds, flavonoids and antioxidant activity studies were evaluated according to the Spearman rank correlation coefficient. The presented correlation graph (Figure 4) shows a strong correlation between the total amount of active phenolic compounds estimated by HPLC analysis and the results of antioxidant ABTS (ρ = 0.89 > 0) and DPPH (ρ = 0.9 > 0). There is also a strong correlation between the total amounts of flavonoids in the samples determined by HPLC analysis and the results of reduction activity tests FRAP (ρ = 0.93 > 0) and CUPRAC (ρ = 0.88 > 0).

### 2.4. Antibacterial Activity

The results of the study showed (Table 3) that the extracts were effective in inhibiting the growth of the tested microorganisms *C. albicans* and *S. aureus*.

The N1 extract showed better activity against *S. aureus* as the MIC of phenolic compounds was 0.491 ± 0.012 mg CAE/g. The weakest effect was observed with the extract N2, whose MIC against *S. aureus* was 0.905 ± 0.033 mg CAE/g. The studied extracts acted more strongly against the fungus *C. albicans* than against *S. auereus*. N1 extract inhibited the fungus *C. albicans* when the MIC of phenolic compounds was 0.394 ± 0.013 mg CAE/g, and the weakest growth of the fungus *C. albicans* was inhibited by N2 extract with a MIC of phenolic compounds of 0.905 ± 0.033 mg CAE/g. The N3 extract showed an effect against *S. aureus* with a MIC of 0.635 ± 0.016 mg CAE/g for phenolic compounds and 0.424 ± 0.014 mg CAE/g against *C. albicans*.

## 3. Discussion

The data of scientific research on the chemical composition and biological activity of propolis that is published in the scientific literature can be applied in food, pharmaceutical and cosmetic industries when creating new products with propolis.

The antibacterial properties of propolis are attributed to phenolic compounds, especially flavonoids, phenolic acids and their esters [28]. Given that the chemical composition of propolis depends on the plants growing in the place of its collection, more and more research is being done on that. The obtained results have shown that Lithuanian propolis had typical phenols of “poplar buds”: flavonoid aglycones (flavones and flavonones), phenolic acids and their esters [28,29]. 

In ethanol extracts of propolis tested from Portugal and Brazil, the total content of phenolic compounds ranged from 29.5 to 137 mg gallic acid eq/g [30], while higher levels of phenolic compounds were detected in propolis extracts from Poland and China, from 150 to 340 mg gallic acid eq/g [28,31]. About 130 mg *p*-coumaric acid eq/g was detected in our 70% ethanol (*v*/*v*) extract, a result that coincides with the literature, which indicates the total amount of phenolic compounds in Lithuanian propolis [30]. We performed a colorimetric method with aluminum chloride to determine the total amount of flavonoids. The spectrophotometric method is applicable to flavonoids only in certain groups and it is appropriate to further develop the trials based on HPLC analysis. In Algerian black poplar buds, the total amount of flavonoids in ethanolic extract reaches about 13.65 mg quercetin eq/g [32], and researchers found that Polish propolis has a total flavonoid content of 18.76 mg quercetin eq/g [33]. In Lithuanian black poplar buds’ extract, we found higher amounts of flavonoids compared to Algerian researchers, around 24.76 mg rutin eq/g, and similar amounts of flavonoids in propolis extract compared to Polish propolis extract [32,33]. Other authors found similar amounts of flavonoids in black poplar buds [34], but no data were found on the total amount of flavonoids in balsamic poplar buds. The obtained results confirmed the earlier description of the researchers’ statements that the amounts of phenolic compounds depend on the geographical region [4,35], greatly influenced by the chosen solvent and extraction technology [36]. Comparing the total amount of phenolic compounds and flavonoids in plant raw material is difficult because there is no international standard for measuring phenolic compounds and flavonoids, and different standards are used in the studies (gallic acid, caffeic acid, *p*-coumaric acid and others for total phenolic compounds, and rutin, quercetin and others for total flavonoids) [37]. 

Typical flavonoids of poplar buds were found in balsam poplar bud extract [3]. Study results of Lithuanian scientists confirmed that the dominant acids in Lithuanian propolis were *p*-coumaric acid and vanillin [38]. During experimental research, it was determined that *p*-coumaric acid dominated in balsam poplar buds, while caffeic acid dominated in black poplar buds. The data of our research were identical with the scientific research of other countries (Great Britain, France, Italy, the Netherlands, Belgium, Russia and Eastern China), which determined that a large amount of caffeic acid was found in black poplar buds [12,18,39]. However, differently than in the published research data, we found that in the analyzed poplar bud extracts, there was a small amount of ferulic acid. Balsam poplar extract had the largest amount of cinnamic acid, which gives an exceptional odor to this extract. It was published in the scientific literature that the dominant acids in European propolis were caffeic, cinnamic and ferulic [40,41,42]. Those acids are not dominant in Lithuanian propolis. The results of the study confirmed that the variety of phenolic acids in propolis depends on the vegetation that predominates in the harvesting area [43,44]. One of such plants is balsam poplar, which is a little more common in Lithuania than black poplar. At the same time, the results of the studies confirmed the data of the scientific literature, that the chemical composition of poplar buds may differ due to diverse phenolic compounds, such as flavonoids, aglycones and their chalcones, and also phenolic acids and their esters [12]. Pinocembrin and galangin are found in many samples of propolis in other countries (propolis from the Northwest of Argentina as a source of antifungal principles) [45,46]. In propolis extract, which we analyzed, the quantity of flavonoids was relatively smaller than the set total amount of compounds. Kaempferol is one of the main compounds which is associated with the anti-inflammatory activity of Brazilian propolis [47], but in the exact extracts that we analyzed, that compound was not found. There was a higher amount of flavonoids found in the balsam poplar bud extract than in other extracts. In the analyzed poplar extracts, salicin was identified, which is found in Salicaceae family trees and shrubs, poplars among them. It is one of the most well-known salicylic compounds, which was discovered in the 19th century [48]. Salicin compounds perform a protective function in the plants to environmental stress and pathogen attacks [49]. Salicin was not identified in propolis and that was probably due to the fact that poplar buds had very little amount of salicin and also because propolis was also collected from plants other than poplars. The results of the study show that most of the bioactive compounds contained in propolis come from tissues and plant liquid secretions [3,35]. Therefore, many of the compounds identified in poplar bud extracts are also found in propolis, only in different amounts.

The active compounds identified in propolis and poplar bud extracts have antibacterial and antioxidant activity [13]. While assessing antioxidant activity, simple, quickly performable, easily repeatable methods were used. All methods confirmed the antioxidant activity of the extracts. The differences in study results when evaluating activity by different methods can be associated with the solubility of radicals used. By using the DPPH method, only antioxidants soluble in organic solvents can be identified. DPPH radical is sensitive to light, oxygen and pH changes, and results are different when different solvents are used [50,51]. By the FRAP spectrophotometric method, only the antioxidant activity of water-soluble, but stable in acidic medium compounds can be determined [52,53,54]. On the basis of data obtained in the course of this study, the tested extracts were identified as effective antioxidants while tested in vitro when their activity was compared with the standard antioxidant, such as ascorbic acid. The results of the tests confirmed the antioxidant activity of *p*-coumaric acid [15,55]. The results confirm published research data that shows that poplar bud extracts have high antioxidant activity [1] due to their chemical composition, caffeic and *p*-coumaric acids included. The results of our research have shown that the antioxidant activity of the tested poplar buds and propolis extracts directly depended on the quantity of active compounds. Strong correlation was determined between the quantity of active compounds and antioxidant activity. A test of antibacterial activity confirmed that propolis was a significant antimicrobial bee product [56,57,58]. The determined MIC of propolis extract was similar to the published data of other researchers [59]. Other researchers have also determined that the action of propolis extract is stronger against *S. aureus* and limited against Gram-negative bacteria [59]. The test results also revealed that poplar bud extracts have a suppressive effect on the growth of the microorganisms tested. Other researchers also determined that poplar bud extract elicited significant antifungal activity against *C. albicans* (MIC = 45.16 µg/mL) [60]. Balsam poplar extract had higher antibacterial activity than black poplar extract, which can be associated with its richer composition of the active compounds. Scientists associate the antimicrobial action of poplar bud extracts with flavonoids and phenolic acids [58,61], since a larger amount of active compounds was determined in balsam poplar extracts that could have conditioned a higher antibacterial activity in comparison with black poplar extract. Propolis occupied an intermediate place between the tested poplar bud extracts. On the basis of the total amount of active compounds and the test results of their antibacterial and antioxidant activity, the extracts can be grouped as N1, N3 and N2. Bioactive compounds from plants are classified according to functional, pharmacological and toxicological effects [27]. The results of our research show that poplar buds are a source of active compounds in propolis, which have antioxidant and antimicrobial actions.

## 4. Materials and Methods

### 4.1. Materials

The raw plant material was collected in the northern part of Lithuania in the spring (March) of 2020, when the maturation of poplar buds began. Fresh poplar buds were dried by the supplier. Dried raw plant material was used for the study. Poplar buds were purchased at Jadvyga Balvočiūtė’s organic herbal farm, and propolis was obtained from Brolių medus.

Rectified ethanol for food purposes, 96.3% (JSC “Vilniaus degtine”, Vilnius, Lithuania), was used. All solvents, reagents and standards used were of analytical grade: acetonitrile (Sigma-Aldrich, Steinheim, Germany), Folin–Ciocalteu’s reagent (Sigma-Aldrich, Buchs, Switzerland), reference standards *p*-coumaric acid (Sigma-Aldrich, Steinheim, Germany), caffeic acid (Sigma-Aldrich, Steinheim Germany), chlorogenic acid (Sigma-Aldrich, Steinheim, Germany), ferulic acid (Sigma-Aldrich, Buchs, Switzerland), vanillic acid (Sigma-Aldrich, Buchs, Switzerland), vanillin (Sigma-Aldrich, Buchs, Switzerland), apigenin (Sigma-Aldrich, Buchs, Switzerland), pinobanksin (Sigma-Aldrich, Buchs, Switzerland), pinocembrin (Sigma-Aldrich, Buchs, Switzerland), galangin (Sigma-Aldrich, Buchs, Switzerland), kaempferol (Sigma-Aldrich, Buchs, Switzerland), salicin (Sigma-Aldrich, Buchs, Switzerland) and cinnamic acid (Sigma-Aldrich, Steinheim, Germany). Purified deionized water used in the tests was prepared with the Milli-Q^®^ (Millipore, Burlington, Massachusetts, USA) water purification system.

### 4.2. Preparation of the Extracts

The raw plant material used in the extraction was dried by the supplier. The raw material was stored in a refrigerator in an opaque paper bag. Poplar buds were crushed before extraction. Extraction was performed in an ultrasonic bath for 60 min, at 35 °C temperature, and 70% ethanol (*v*/*v*) was used as the extractant with a ratio of raw material and solvent 1:10. After extraction, extracts were stored in the refrigerator for 24 h at a 5 °C temperature. All extracts were filtered through ashless filter paper (retention 8–12 µm, diameter 90 mm, ash content 0.007%) [15].

### 4.3. Determination of Total Phenolic Content

Propolis, balsam poplar and black poplar buds’ extracts were evaluated spectrophotometrically using Folin–Ciocalteu reagent, according to Singleton et al. [62], with a few modifications. Extract sample (150 µL) was mixed with Folin–Ciocalteu reagent (750 µL), and after 3 min, 7.5% sodium carbonate was added (600 µL). Samples were incubated for 30 min at room temperature (RT). Total amount of phenolic compounds was evaluated spectrophotometrically at 760 nm (Agilent Technologies 8453 UV–Vis, Santa Clara, CA, USA). Results were expressed as mg of *p*-coumaric acid equivalent per gram of dry weight (mg CAE/g).

### 4.4. Determination of Total Flavonoids Content

Flavonoid determination was performed based on Woisky and Sakatin’s method [63] with a few modifications. Balsam poplar buds and propolis extracts (500 µL) were mixed with 96.6% ethanol (1500 µL), 10% aluminum chloride (Sigma–Aldrich, Saint Quentin-Fallavier, France) (100 µL), 33% acetic acid (100 µL) and distilled water was added (2800 µL) to mixtures. Reaction mixtures were incubated for 30 min at RT. The absorbance at 407 nm was measured. Results were expressed as mg of rutin equivalent per gram of dry weight (mg RE/g).

### 4.5. High-Performance Liquid Chromatography (HPLC)

The identification of the predominant active compounds was performed by high-performance liquid chromatography (Table 2). A Waters 2695 chromatographic system with a Waters 996 diode array detector and an ACE 5C18 chromatography column (250 × 4.6 mm) was used. Data was processed by Empower 2 Chromatography Data Software. The eluent system consists of 100% acetonitrile and 1% trifluoroacetic acid, and the 1% trifluoroacetic acid—eluent A, 100% acetonitrile—eluent B, elution program was used as follows: from 5% to 15% B at 0–8 min, from 15% to 20% B at 8–30 min, from 20% to 40% B at 30–48 min, from 40% to 50% B at 48–58 min, from 50% to 50% B at 58–65 min, from 50% to 95% B at 65–66 min, from 95% to 95% B at 66–70 min, from 95% to 5% B at 70–71 min. Injection volume 10 µL, column temperature 25 ° C, mobile phase flow rate 1 mL/min, flow time 81 min. The compounds in the sample were identified by the retention time of the analytes and reference substances present and the UV absorption from 300 to 360 nm [38]. Reference compounds: *p*-coumaric acid (RT = 19.669, R^2^ = 0.9999), caffeic acid (RT = 14.084, R^2^ = 0.9999), salicin (RT = 9.042, R^2^ = 0.9999), apigenin (RT = 47.706, R^2^ = 0.9999), galangin (RT = 58.636, R^2^ = 0.9998), kaempferol (RT = 48.581, R^2^ = 0.9999), pinobanksin (RT = 47.158, R^2^ = 0.9999), pinocembrin (RT = 57.967, R^2^ = 0.9998), vanillin (RT = 17.488, R^2^ = 0.9999), vanillic acid (RT = 13.456, R^2^ = 0.9999), cinnamic acid (RT = 43.246, R^2^ = 0.9999), ferulic acid (RT = 21.893, R^2^ = 0.9999), chlorogenic acid (RT = 11.538, R^2^ = 0.9999). The extracts were diluted 10 times with 70% ethanol (*v*/*v*) before HPLC analysis. The results were presented as the mean of three measurements, *p* < 0.05.

### 4.6. Antioxidant Activity by ABTS, DPPH, FRAP and CUPRAC Methods

For the antioxidant activity, original ethanolic extracts N1, N2 and N3 were used.

The antiradical activity of the extracts was determined by the ABTS method, with certain modifications according to the methodology of Yim et al. [64]. Prepared stock solution of ABTS was kept in the dark for 16 h, until the oxidation-reduction reaction takes place and ABTS is formed. Working ABTS solution was prepared by diluting stock solution with purified water until absorbance reaches 0.8 at 734 nm. 3 µL of poplar buds extracts and propolis extracts was mixed with 3000 µL of ABTS working solution (Sigma-Aldrich, Oakville, ON, Canada). Samples were incubated for 30 min at RT. The absorbance was measured with a spectrophotometer at a 734 nm wavelength.

The antiradical activity of the extracts by the DPPH method was performed with certain modifications according to Yim et al.’s methodology [64]. The working solution of DPPH was prepared from the stock solution, diluting it with purified water until the working solution reaches an absorbance of 0.8 at 517 nm. 10 µL of poplar buds extracts and propolis extracts was mixed with 3000 µL of DPPH working solution. Samples were incubated for 30 min at RT. The absorbance was measured with a spectrophotometer (Agilent Technologies 8453 UV-Vis, Santa Clara, CA, USA) at a 517 nm wavelength. 

FRAP reducing activity was assessed based on Raudonės et al.’s methodology [65] with some modifications. Working solution of FRAP was prepared from 300 mmol/L sodium acetate buffer solution, 10 mmol/L TPTZ solution and 20 mmol/L FeCl_3_ × 6H_2_O aqueous solution in a ratio of 10:1:1. 10 µL of poplar buds extract was mixed with 3000 µL of FRAP working solution. Samples were incubated for 30 min at RT. The absorbance was measured with a spectrophotometer at a 593 nm wavelength. 

CUPRAC reducing antioxidant capacity was evaluated by Apak et al.’s methodology [66] with a few modifications. A working solution of CUPRAC was obtained by mixing 10 mmol/L CuCl_2_, 1 mmol/L CH_3_COONH_4_ buffer solution, pH 7, and 7.5 mmol/L neocuproin ethanol in a ratio of 1:1:1. 5 µL of the test extract was mixed with 3 mL of CUPRAC working solution. Samples were incubated for 30 min at RT. The absorbance was measured with a spectrophotometer at a 450 nm wavelength.

Calibration graphs were prepared by using trolox standard solutions. The results were expressed as µmol/g trolox equivalent (µmol TE/g).

### 4.7. Antimicrobial Activity

Original ethanol extracts N1, N2 and N3 were used. The study was performed on the basis of Ph. Eur. 2002 01 01, 2.6.12, and the microbiological test was performed under aseptic conditions. The effect of the studied extracts was investigated on *S. aureus* ATCC 25923 (TSB—Tryptic Soy Broth medium, “Sigma–Aldrich”, France) and *C. albicans* ATCC 60193 (YPD—Yeast Peptone Dextrose medium, “Sigma–Aldrich”, France). The minimal inhibitory concentration was assessed in the study (MIC). *S. aureus* and *C. albicans* colonies were dissolved in tubes containing normal saline to obtain inoculum suspensions. Inoculum suspensions were transferred to 96-well plates containing samples of various dilutions, and all extracts were diluted with the same ratio, from 100 to 800 times. The MIC value was indicated as the lowest concentration of tested extracts that inhibited the growth of *S. aureus* or *C. albicans* after incubation. *S. aureus* and *C. albicans* were incubated in a 37 °C thermostat for 24 h. Ampicilinum (Sigma–Aldrich) and streptomycin (Sigma–Aldrich) were used as a positive control, and 70% ethanol was used as a negative control, with the same dilution ratio as extracts N1, N2 and N3 [38].

### 4.8. Statistical Analysis

Results were presented as the mean and standard deviation of three measurements. For independent measurements, the nonparametric Kruskal–Wallis test was used. For variables, for which the assumption of normality is not satisfied, the correlation was calculated based on the Spearman correlation coefficient. All data were evaluated and presented using OriginPro^®^2021 (OriginLab, Northampton, MA, USA) and IBM SPSS Statistics 27 (SPSS Inc., Chicago, IL, USA). Results are considered statistically significant when *p* < 0.05.

## 5. Conclusions

Different poplar species growing in Lithuania are characterized by different compositions of active compounds and their amounts, and these differences are reflected in the composition of propolis, which includes bud exudates. Balsamic poplar bud extracts have a higher content of tested compounds compared to black poplar and propolis extracts. *P*-coumaric acid is the predominant phenolic acid in balsamic poplar buds and propolis extracts. The study showed that poplar buds and propolis extracts are important as antioxidants and antimicrobial agents. The analysis of the results provided new data about Lithuanian poplar buds’ chemical composition and biological activity and some extra data about the similarities and differences between propolis and poplar buds’ chemical composition and biological activity. It also provided some information about possible applications of this raw material in the production of food, cosmetic and pharmaceutical products.

## Figures and Tables

**Table 1:** Quality results of poplar buds and propolis extracts (N1—*P. balsamifera* buds extract, N2—*P. nigra* buds extract, N3—Propolis extract). Total phenolic compounds mg CAE/g ± SD of dry weight, mean, *n* = 3. Total flavonoids mg RE/g of dry weight, mean, *n* = 3.

Series	Extract Appearance	Total Phenolic Compounds	Total Flavonoids
N1	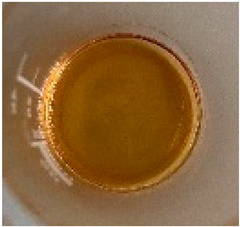	196.81 ± 10.42 ^a^	46.13 ± 1.12 ^a^
N2	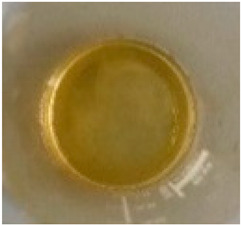	95.02 ± 1.93 ^b^	24.76 ± 0.97 ^b^
N3	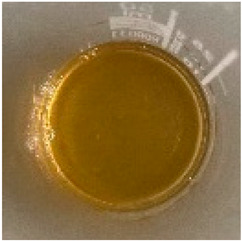	127.14 ± 2.33 ^c^	18.79 ± 0.74 ^c^

^a,b,c^ Series sharing superscript letters showed statistical difference between extracts N1, N2 and N3.

**Table 2:** The amounts of active compounds in the poplar buds and propolis extracts (mean mg/g ± SD of dry weight, *n* = 3). CoV (%): Coefficient of variation.

Active Compounds	*P. balsamifera* 70% ETOH (mg/g ± SD)	CoV (%)	*P. nigra* 70% ETOH (mg/g ± SD)	CoV (%)	Propolis 70% ETOH (mg/g ± SD)	CoV (%)	Pairwise Difference *
1. Salicin	0.2855 ± 0.0100	3.52	0.8188 ± 0.0163	1.99	-	-	ab
2. Chlorogenic acid	0.3496 ± 0.0090	2.58	0.2557 ± 0.0099	3.85	-	-	ab
3. Vanillic acid	0.0026 ± 0.0001	2.92	0.0236 ± 0.0004	1.64	0.4924 ± 0.0099	2.03	abc
4. Caffeic acid	0.5993 ± 0.0700	1.29	1.6818 ± 0.0106	0.64	0.4850 ± 0.0041	0.82	abc
5. Vanillin	-	-	0.0141 ± 0.0009	6.04	4.3266 ± 0.0109	0.44	bc
6. *P*-coumaric acid	13.5461 ± 0.3940	0.29	0.7086 ± 0.0118	1.66	14.4116 ± 0.0503	0.35	abc
7. Ferulic acid	0.0077 ± 0.0001	2.02	0.0242 ± 0.0005	2.27	0.4267 ± 0.0066	1.42	abc
8. Cinnamic acid	10.6687 ± 0.5220	0.49	-		0.3924 ± 0.0153	3.95	ac
9. Pinobanksin	1.9218 ± 0.0268	1.39	0.1495 ± 0.0058	3.98	0.5336 ± 0.0132	2.48	abc
10. Apigenin	0.5139 ± 0.0103	2.01	0.1666 ± 0.0071	4.28	0.2707 ± 0.0074	2.74	abc
11. Kaempferol	-	-	-	-	-	-	
12. Pinocembrin	0.5055 ± 0.0107	2.12	1.0743 ± 0.0179	1.67	-	-	ab
13. Galangin	8.7581 ± 0.0119	0.14	0.1679 ± 0.0077	4.60	0.1139 ± 0.0051	4.61	abc
Total flavonoids (mg/g)	11.6993		1.5583		0.9182		abc
Total phenolic acids (mg/g)	25.1738		2.6939		16.2081		abc
Total amount of identified compounds (mg/g)	37.1587		6.6433		21.4529		abc

* For each active compound, lowercase letters indicate which extracts (a for *P. balsamifera*, b for *P. nigra*, c for Propolis) showed a pairwise statistically significant difference.

**Table 3:** MIC of *P. balsamifera* buds extract—N1, *P. nigra* buds extract—N2 and Propolis extract—N3, mg CAE/g ± SD (dry weight), mean, *n* = 3, *p* < 0.05.

Serie	*S. aureus* MIC	*C. albicans* MIC
N1	0.491 ± 0.012	0.394 ± 0.013
N2	0.905 ± 0.033	0.905 ± 0.033
N3	0.635± 0.016	0.424 ± 0.014

## Data Availability

Data available in a publicly accessible repository.
